# Investigation on the Residual Stresses and Fatigue Performance of Riveted Single Strap Butt Joints

**DOI:** 10.3390/ma13153436

**Published:** 2020-08-04

**Authors:** Jintong Liu, Anan Zhao, Zhenzheng Ke, Zhiqiang Li, Yunbo Bi

**Affiliations:** 1 State Key Laboratory of Fluid Power and Mechatronic System, College of Mechanical Engineering, Zhejiang University, Hangzhou 310027, China; liujintong@zju.edu.cn; 2 Key Laboratory of Advanced Manufacturing Technology of Zhejiang Province, College of Mechanical Engineering, Zhejiang University, Hangzhou 310027, China; kzzcaen@zju.edu.cn (Z.K.); 21725021@zju.edu.cn (Z.L.); 3 Aviation Industry Corporation of China Xi’an Aircraft Industry (Group) Limited Company, Xi’an 710089, China; zhaoaa@avic.com

**Keywords:** riveted butt joint, residual stress, fatigue performance, finite element modeling

## Abstract

In aircraft manufacturing, riveting is one of the most important connection ways to fasten the sheet metal parts. The riveted single strap butt joints are mainly used in the load-bearing components of the aircraft such as the fuselage and wing panels. The connection quality and fatigue performance of the riveted joints directly affect the reliability and safety of the aircraft. In this paper, under the assumption of constant temperature, the fatigue strengthening mechanism of interference-fit riveting is introduced based on elastic-plastic mechanics and fracture mechanics. On this basis, the finite element (FE) models of the riveted single strap butt joints with various strap thickness and rivet sizes/arrangements are established. The residual stresses distribution around the riveted hole is analyzed. Furthermore, the fatigue tests of the riveted single strap butt joints with cyclic loading are carried out. The experimental results verified the correctness and effectiveness of the simulation model. Finally, the conclusion is drawn that increasing rivet size and strap thickness within the allowable weight range can improve the fatigue performance of the riveted single strap butt joints. The knowledge could be used to guide the structural design and optimization of the riveted butt joints against fatigue.

## 1. Introduction

The low-density components made of aluminum or titanium alloys are widely used in the aircraft industries to reduce the weight of the product, without compromising quality and performance [1]. Subsequently, the joining technologies of sheet metal parts have become a key factor affecting the assembly quality of aircraft. Among various mechanical connection methods, riveting plays the most important role in aircraft manufacturing because of its stable, easy trouble-shooting, convenient quality inspection and low cost [2,3]. Stress Load and cyclic temperature load have important influence on fatigue performance of a riveted joint. In this paper, the effect of stress load is analyzed under the assumption of constant temperature.

**Figure 1:**
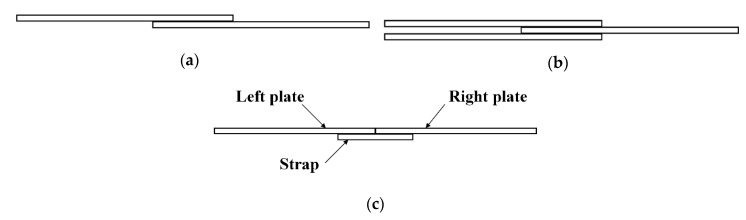
Several typical riveted joints configurations: (**a**) single-lap joint; (**b**) double-lap joint; and (**c**) single strap butt joint.

Generally, there are several typical riveted joints, such as a single-lap joint, a double-lap joint, and a single-trap butt joint, as shown in Figure 1 [4]. The lap joint has a shorter connection area and is lighter in weight because there are no excess connection parts. However, the overlapped panels are easy to sag, which leads to stress concentrations and affect the fatigue performance of the joints. In addition, the high-stress zone in the lap joint is on the panels, once the cracks appear, it is more difficult to maintain and control the propagation of the cracks. On the other side, the butt joint is heavier than the lap joint due to the use of straps. However, the butt joint can avoid sagging of the panels and has fewer stresses concentration. The high-stress zone in the butt joint is on the straps, which is easier to repair when cracks appear [5].

A key step for understanding the fatigue performance of the riveted joints is to analyze the riveting process. A great deal of interest has been focused on the modeling and analysis of the riveting process by researchers. Zhang et al. [4] studied the riveting process based on mathematical modeling and simulating to analyze the deformation of thin-walled sheet-metal parts. Aman et al. [6] built finite element models to study the effect of riveting sequence, the distance between rivets, and the gap between sheets on the quality of riveted lap joints. A combination of riveting process parameters is found to minimize the residual stress in sheets and rivets. Chang et al. [7] built a 3D finite element (FE) model for the riveting process and validated it by experiments. The study found that the riveting sequence has a significant effect on the distribution of riveting deformation. Furthermore, the riveting induced residual stress distribution around the riveted hole area also attracted the attention of researchers. Li et al. [8] developed a two-dimensional axisymmetric finite element model of fuselage lap joints. Non-linear and nonhomogeneous distribution properties for the residual stress was displayed. Li et al. [9] developed a three-dimensional numerical technique to study the residual stress and strain as well as their variations that occur during the riveting process. Manes et al. [10] investigated the effects of squeeze force, the clearance, the rivet length, and the clamping angle in the stress field of the riveted joints by numerical technique. The results demonstrated that the squeeze force is the key parameter in the characterization of the stress field. Elajrami et al. [11] analyzed the residual stress distribution in the riveted hole of aluminum alloy Al2024-T3 induced by a tapered pin. The results show that the residual stresses vary through the material depth, are strong at the exit face and moderate at the entrance face and maximal in mid-depth.

The fatigue life of the riveted joints mainly depends on the coupling effect of residual stress induced by the riveting process and the external load during ground-air-ground pressurization cycles [12]. Therefore, the fatigue behavior and cracks propagation of riveted joints is a momentous aspect of riveting research. Rans et al. [13] employed the fractographic techniques to evaluate postmortem the influence of the riveting squeeze force and resulting residual stresses on crack behavior for the riveted lap joint. The results of fatigue tests show that the fatigue life increased with increasing riveting squeeze force. Muller [14] made an extensive investigation on riveted lap joints, including theoretical and experimental analysis. The 3D residual stress distribution was studied by FE calculations and the results agree well with observations on fatigue life enhancement in experiments. The studies indicated that the squeeze force has a significant effect on the location of fatigue crack nuclei and crack growth through or around the rivet hole. Armentani et al. [15] conducted fatigue tests for 200 single lap multiple-riveted joint specimens and the fatigue lives and critical crack sizes for all specimens were analyzed. The results showed that the higher the applied stress, the more through-hole cracking appeared. Skorupa et al. [16] investigated the impact of sheet thickness, rivet type and squeeze force on the fatigue response of the riveted lap joint. It was observed that the fatigue cracks always start in one of the end rivet rows of the loaded sheet. Besides, the establishment of the fatigue life prediction model has important reference significance for the practical application of riveted joints. Skorupa et al. [17] developed a fatigue life prediction model for riveted lap joints, in which the rivet hole expansion and the load transfer distribution are unambiguously characterized by measurable quantities. The model is validated by 80 fatigue tests on aluminum alloy. De Rijck et al. [18] investigated the relation between squeeze load and the rivet head dimension during controlled squeezing. Then the quality control of the riveting process concerning the fatigue performance of riveted joints can be achieved. Leonetti et al. [19] proposed a system reliability model for estimating the probabilistic fatigue life of riveted shear connections. The model allows considering multiple site cracks.

However, most of the existing research is based on the lap joints, while the research of the riveted butt joint is relatively few. Maljaars et al. [20] presented a theoretical fatigue strength prediction model for riveted double-covered butt joints. The study indicated that the plate width over rivet diameter radio and the surface finish has a large effect on the strength. Citarella et al. [21] carried out a multi-site-damage crack growth simulation of a cracked riveted butt-joint. The equivalent crack length is proposed for a 2D analysis and the simplified bidimensional analyses are validated by fatigue test data. Galatolo et al. [22] presented a combined experimental and numerical analysis of the residual strength of a butt-joint panel with multiple-site-damage. A crack propagation model accommodated for crack growth resistance, crack link-up and in particular restart after link-up was proposed. In fact, the effects of rivet size/arrangement and strap geometry on the fatigue life of the riveted butt joint are still scarcely understood. Moreover, the relationship between the residual stress distribution and the fatigue performance of riveted butt joint has not been revealed.

In this paper, under the assumption of constant temperature, the residual stresses of riveted single strap butt joints are numerically studied with various rivet sizes and strap thicknesses. Then, fatigue tests with cyclic loading are carried out to investigate the fatigue life of the riveted butt joint specimens with various parameters. The optimal structural design parameters of the riveted butt joint are finally determined. The remainder of the paper is organized as follows: In Section 2, a brief introduction of interference riveting is provided. The residual stresses distribution around the riveted hole and the fatigue strengthening mechanism are studied. In Section 3, the numerical simulation is performed, the details of the FE model are described. The driven head geometries and the residual stresses with various rivet sizes and strap thicknesses are studied. In Section 4, fatigue tests are conducted with the automatic drilling and riveting system to verify the simulation results. The experimental results and discussion are presented. At last, the conclusion and future works are summarized in Section 5.

## 2. Theoretical Basis

### 2.1. Interference-Fit Riveting

**Figure 2:**
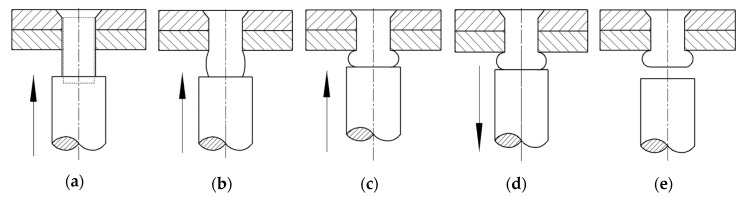
Interference-fit riveting process. (**a**) Rivet starts to deform and fill the hole, but not yet contact with plates; (**b**) Rivet contacts plates and the driven head starts to form; (**c**) The driven head is formed and rivet is in full contact with plates; (**d**) The riveting bar retreats and the spring-back of the riveted joint occurs; and (**e**) Riveting bar separates from rivet; the riveting process is over.

Interference-fit riveting can improve the strength of mechanical connection through interference fit between rivet and plates. It can increase the fatigue life while keeping the current structure form and weight of the components [23]. The process of interference-fit riveting can be divided into five stages: rivet hole filling, rivet and plates contacting, driven head forming, riveted joint springing-back, and riveting ending, as shown in Figure 2.

**Figure 3:**
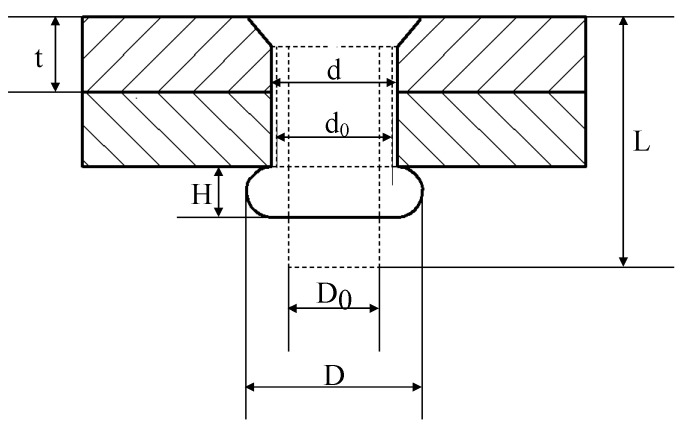
The final shape of the riveted joints. *t*: Plate thickness; $d_{0}$: Rivet hole diameter before riveting; *d*: Rivet hole diameter after riveting; $D_{0}$: Rivet diameter; *L*: Rivet length; *D*: Driven head diameter; *H*: Driven head height.

The shape of the riveted joint after riveting is illustrated in Figure 3. The relative interference has a great influence on the residual stress and fatigue performance of the riveted joint, and its definition and calculation method are described.

With the following assumptions, the relationship of rivet volume before and after riveting can be derived as Equation (1).

1. Assume that the rivet volume is constant during the riveting process.
2. Assume that the deformation of the rivet expands uniformly along the direction of the rivet bar.

(1) $$\frac{\pi }{4}LD_{0}^{2}\approx \frac{\pi }{2}td^{2}+\frac{\pi }{4}HD^{2},$$

where L represents the rivet length, $D_{0}$ is the rivet diameter, t represents the plate thickness, d is the rivet hole diameter after riveting, and H and D represent the height and diameter of the driven head, respectively. According to Equation (1), the relative interference can be expressed as:

(2) $$\Delta =\frac{d-d_{0}}{d_{0}}\times 100\%,$$

where $d_{0}$ represents the rivet hole diameter before riveting and $d-d_{0}$ is the absolute interference, which can be represented as:

(3) $$d-d_{0}=\sqrt{\frac{LD_{0}^{2}-HD^{2}}{2t}}-d_{0},$$

### 2.2. Fatigue Strengthening Mechanism of Rriveted Joints

During the riveting process, the elastic-plastic deformation is produced on the rivet and plates and thus the residual stress appears near the riveted hole area. According to the mechanism of hole cold extrusion strengthening, the compressive residual stress in the hole wall can effectively reduce the local stress concentration and improve the fatigue life of riveted joints [24]. For further explanation, the stresses distribution around the riveted hole will be theoretically analyzed in this section.

**Figure 4:**
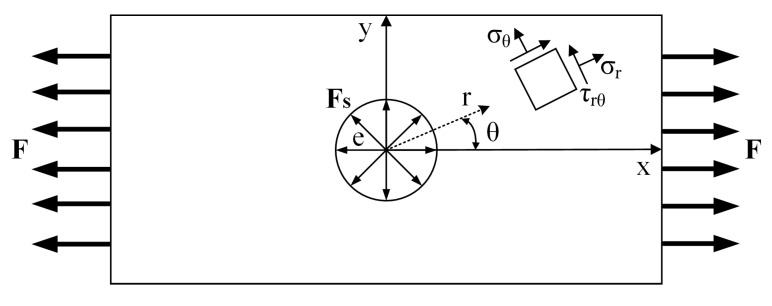
Schematic diagram of the polar coordinate on the plate surface. *F*: Tensile force; $F_{s}$: squeezing force on the hole wall; e: Rivet hole radius; $\sigma_{\theta }$: Circumferential stress; $\sigma_{r}$: Radial stress; $\tau_{r\theta }$: Shear stress; r: Radius of polar coordinate system; and θ: Angle of polar coordinate system.

Firstly, a polar coordinate is established on the plate surface, as shown in Figure 4.

According to the elastoplastic mechanics [24], the circumferential, radial and shear stress values of a microelement on the plate can be expressed as:

(4) $$\begin{array}{c} \sigma_{r}=\frac{1}{2}F\left[1-\frac{e^{2}}{r^{2}}+\left(1-\frac{4e^{2}}{r^{2}}+\frac{3e^{4}}{r^{4}}\right)cos2\theta \right]\\ \sigma_{\theta }=\frac{1}{2}F\left[1+\frac{e^{2}}{r^{2}}-\left(1+\frac{3e^{4}}{r^{4}}\right)cos2\theta \right]\\ \tau_{r\theta }=-\frac{1}{2}F\left(1+\frac{2e^{2}}{r^{2}}-\frac{3e^{4}}{r^{4}}\right)sin2\theta \end{array}\},$$

where $\sigma_{r}$, $\sigma_{\theta }$, and $\tau_{r\theta }$ represent the radial stress, circumferential stress, and shear stress, respectively. F represents the tensile force, $F_{s}$ represents the squeezing force on the hole wall, e represents the rivet hole radius. r and θ represent the radius and angle of the polar coordinate system, respectively.

At the beginning of the interference-fit riveting process, the squeezing force $F_{s}$ on the hole wall is small, only elastically deform occurs on the plate. The stress components around the hole in this stage are shown in Equation (5):

(5) $$\begin{array}{c} \sigma_{r}=-F\frac{e^{2}}{r^{2}}\\ \sigma_{\theta }=F\frac{e^{2}}{r^{2}}\\ \sigma_{z}=\mu \left(\sigma_{r}+\sigma_{\theta }\right)=0 \end{array}\}$$

where $\sigma_{z}$ is perpendicular to the plate and is regarded as 0.

According to Equation (5), when r=e, there will be $\sigma_{r}=-\sigma_{\theta }=-F$. At this time, both $\sigma_{r}$ and $\sigma_{\theta }$ are determined by squeezing force $F_{s}$, and with the increase of $F_{s}$, the circumferential stress $\sigma_{\theta }$ is tensile and the radial stress $\sigma_{r}$ is compressive.

As the riveting bar continues to feed, the squeezing force $F_{s}$ on the hole wall increases and the materials around the hole begin to yield. According to Tresca’s yield condition:

(6) $$\sigma_{\theta }-\sigma_{r}=\sigma_{s},$$

where $\sigma_{s}$ is the yield stress. Substitute Equation (5) into Equation (6), and the following equation is obtained:

(7) $$F=\frac{1}{2}\frac{r^{2}}{e^{2}}\sigma_{s},$$

The materials at the edge of the hole will yield, and the squeezing force at this time is equal to the elastic limit load:

(8) $$F_{s}=\frac{1}{2}\sigma_{s},$$

**Figure 5:**
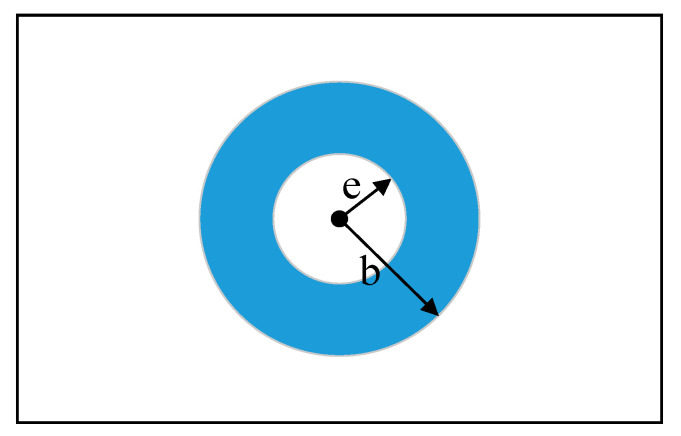
Elastic and plastic deformation zones around the rivet hole. e: Inner radius of the plastic zone and b: Outer radius of the plastic zone.

When the squeezing force $F_{s}$ is larger than $\frac{1}{2}\sigma_{s}$, plastic deformation will occur around the hole area. As shown in Figure 5, assume the radius of the plastic zone is b, then the annular region of e≤r≤b is the plastic deformation zone, while the r≥b region is the elastic deformation zone.

The equilibrium equation for the plastic deformation zone is:

(9) $$\frac{d\sigma_{r}}{dr}+\frac{\sigma_{r}-\sigma_{\theta }}{r}=0,$$

substitute Equation (6) into Equation (9), and we got:

(10) $$\sigma_{r}=\sigma_{s}lnr+A,$$

where A is the integral constant. According to the boundary condition, when r=e, then:

(11) $$\sigma_{r}=-F+\sigma_{s}ln\frac{r}{e}=-F,$$

substitute Equation (11) into Equation (6), then:

(12) $$\sigma_{\theta }=\sigma_{r}+\sigma_{s}=-F+\sigma_{s}\left(1+ln\frac{r}{e}\right)=\sigma_{s}-F,$$

According to Equations (11) and (12), when r=e, the radial stress $\sigma_{r}=-F$, which is a compressive stress and proportional to the squeezing force p. At this time, the circumferential stress $\sigma_{\theta }=\sigma_{s}-p$, which means it not only related to the squeezing force F but also the yield stress $\sigma_{s}$. When $p<\sigma_{s}$, the circumferential stress is tensile; when $F>\sigma_{s}$, the circumferential stress is compressive.

Based on the analysis of stresses distribution around the rivet hole area on the plate, it could be known that with the increase of squeezing force $F_{s}$, there are two interference fit stages between rivet and plates: elastic stage and elastic-plastic stage. In these two stages, the radial stresses $\sigma_{r}$ is always compressive stresses, while the circumferential stresses $\sigma_{\theta }$ changes from tensile stresses to compressive stresses. When the circumferential stresses $\sigma_{\theta }$ is compressive, the stresses amplitude at the hole edge can be effectively reduced, the crack initiation can be delayed, and the fatigue life of riveted joints can be increased [24].

## 3. Simulation Study

In this section, several FE models of the riveted single strap butt joints with various rivet sizes/arrangements and strap thicknesses are established. The effects of these variables on the residual stresses of the riveted joints under constant temperature are analyzed, which provides an optimized basis for improving the fatigue life of riveted single strap butt joints.

### 3.1. Finite Element Modeling

Compared with the actual riveting process, the finite element simulation is still ideal, so the following assumptions need to be made:

1. During the riveting process, the force and displacement loads are applied continuously without interruption.
2. The interior of the rivet and plates are continuous, and there is not gap in the deformed body during riveting.
3. The physical properties of the same component are all the same and do not change with the change of coordinates.
4. The internal stresses of rivet and plates are zero before riveting, and the residual stresses after riveting are all caused by external forces such as squeezing force and tensile force.
5. The effects of temperature and gravity are ignored.

A three-dimensional FE model of the riveted single strap butt joint is built with Abaqus 6.14. The modeling steps are as follows:

● Geometric Model

**Figure 6:**
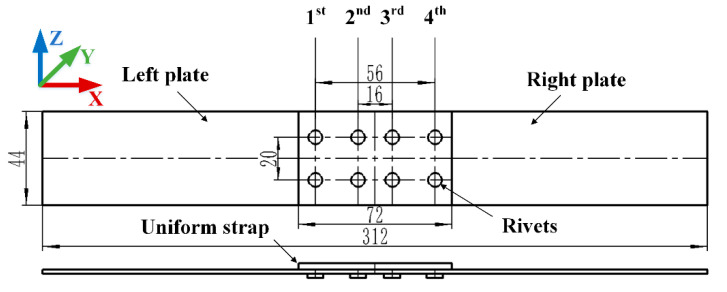
Schematic diagram of riveted single strap butt joint. The strap thickness and the sizes of riveting in four columns are two variable parameters.

As shown in Figure 6, the geometric model of the riveted single strap butt joint consists of five main parts: rigid punch, left plate, right plate, strap, and rivets. The size of the plate is 156 mm in length, 44 mm in width, and 2 mm in thickness. The size of the strap is 72 mm in length, 44 mm in width, and the thicknesses are 2 mm, 2.5 mm and 3 mm, respectively. There are four columns of YSA622-100° rivets and their sizes vary according to the strap thickness. The rivet pitches are 20 mm. There are three kinds of thickness of the strap, and under each thickness, there are three kinds of rivets sizes/arrangements, so there is a total of nine groups. The specific grouping arrangement is shown in Table 1. The group names are set as “strap thickness-rivet diameter of first column-rivet diameter of the second column”, the third and fourth columns of rivets are symmetrical to the first two columns.

● Material Properties

The materials of the strap, left and right plates are 2024-T3 aluminum alloy. The rivets are made of 2117-T4 aluminum alloy. In the FE simulation, an isotropic hardening behavior was assumed for both the rivet and sheet materials. The power exponent hardening constitutive model is employed to describe the stress–strain relationships of the materials. Usually, the material constants *C* and *r* were calculated by substituting the uniaxial tensile test data into $\sigma_{true}=C\cdot \varepsilon_{true}^{r}$, where $\sigma_{true}$ is the true stress, $\varepsilon_{true}$ is the true strain. The material properties are shown in Table 2 and Table 3. 

● Mesh Selection

**Figure 7:**
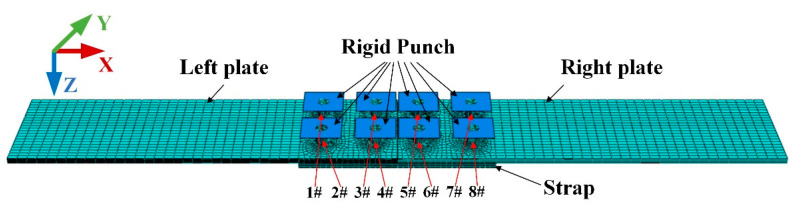
The finite element model of riveted single strap butt joint. The punches are set as rigid bodies and 1#–8# are the numbers of the rivets.

As shown in Figure 7, the hexahedral elements are used for the 3D model. The meshing of riveted joints is generated with reduced integration eight-node solid continuum element C3D8R, which can avoid the problem of shear locking under large deformation. The meshing size is 0.3 for rivets, 1.1 for strap, and 2.8 for plates. Moreover, as shown in Figure 6, the meshing of areas near the rivet hole is further encrypted. Take group 2-5-4 as an example, the total number of mesh elements for the riveted butt joint model is 154,750.

● Analysis Steps

According to the riveting process introduced in Section 2.1, two analysis steps are set for a single rivet: punch down (stage 1 to stage 3) and retract (stage 4 to stage 5). The riveting sequence is based on the principle of “first outside and then inside” to reduce the deformation of the plates. Therefore, the riveting sequence is 1#–8#–2#–7#–3#–6#–4#–5# as shown in Figure 7. So, there are 16 steps in the riveting process period. After that, the 17th step is applying tensile surface traction on the clamping area on the right plate, the direction is *X*-positive. The time of each analysis step is 1 ms.

● Load and Boundary Conditions

The press riveting is controlled by displacement in the FE model. The direction is Z-positive and its value $L_{d}$ can be determined according to the principle of volume conservation of rivet before and after riveting [4]:

(13) $$L_{d}=l-\left(t_{1}+t_{2}-H\right)-\frac{d^{2}l_{2}-d_{1}^{2}\left(t_{1}+t_{2}-H\right)}{D^{2}},$$

where l is the length of the rivet shank, $t_{1}$ and $t_{2}$ are the thickness of the left and right plates, respectively, H is the height of rivet head, D is the average diameter of the driven head, d is the rivet diameter, and $d_{1}$ is the hole diameter.

The ideal diameter of the driven head is D=1.5×d [25]. Then the displacement loads for different sizes of straps and rivets are obtained from Equation (13) and shown in Table 4.

Force control is adopted in the tension analysis step. The force value is determined as:

(14) $$F_{max}=0.7F_{b},$$

where $F_{max}$ is fatigue load. $F_{b}$ is static tensile failure load which is obtained by static tensile test. Then the maximum tangential stress on the surface of clamping area σ can be calculated as:

(15) $$\sigma =\frac{F_{max}}{S},$$

where S is the clamping area.

**Figure 8:**
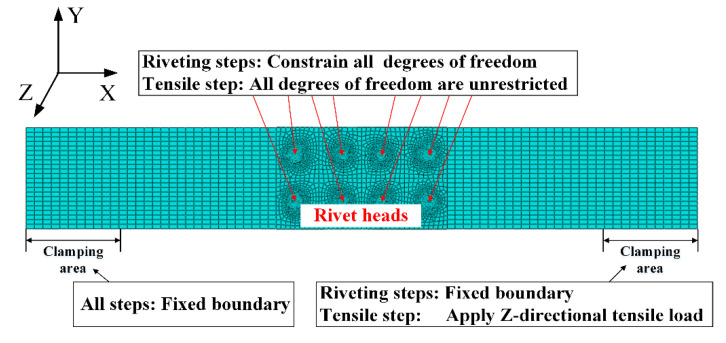
The boundary conditions in the FE model.

The boundary condition in the FE model is shown in Figure 8. All degrees of freedom of the rivet heads are constrained in the riveting steps, then they are inactive in the tensile step. The degrees of freedom in X-direction of plates are constrained during the riveting process of each rivet. After the riveting process, the X-direction of the right plate is set free in tensile step for the application of tension load.

● Contact and Friction Settings

There are several surface interactions in the model, such as:

(a) the contact between the lower surface of punch and the end face of rivet;
(b) the contact between the cylindrical surface of the rivet and the hole wall of riveted hole;
(c) the contact between the cylindrical surface of the driven head and the surface of the plates;
(d) the contact between the side faces of the left and right plate; and
(e) the contact between the strap surface and plates surfaces.

All of these surface interactions are defined by the general contact model which is built in the software. The friction coefficient is set to 0.2 for all the contact interfaces.

### 3.2. Simulation Results

#### 3.2.1. Analysis of Driven Head Geometries

The driven head diameter is an important evaluation index for riveting quality, which can be used to verify the correctness of the FE model.

The driven head diameters and heights after riveting are shown in Table 5. The results show that all the driven head geometries meet the technical requirements. Therefore, the established FE model can effectively simulate the riveting process, the results have good accuracy.

**Figure 9:**
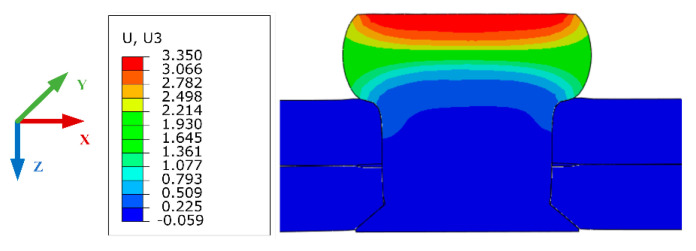
Deformation of 1# rivet in group 2-5-4 in Z-direction.

Take 1# rivet in group 2-5-4 as an example, the deformation of rivet in Z-direction is shown in Figure 9.

#### 3.2.2. Analysis of Interference

**Figure 10:**
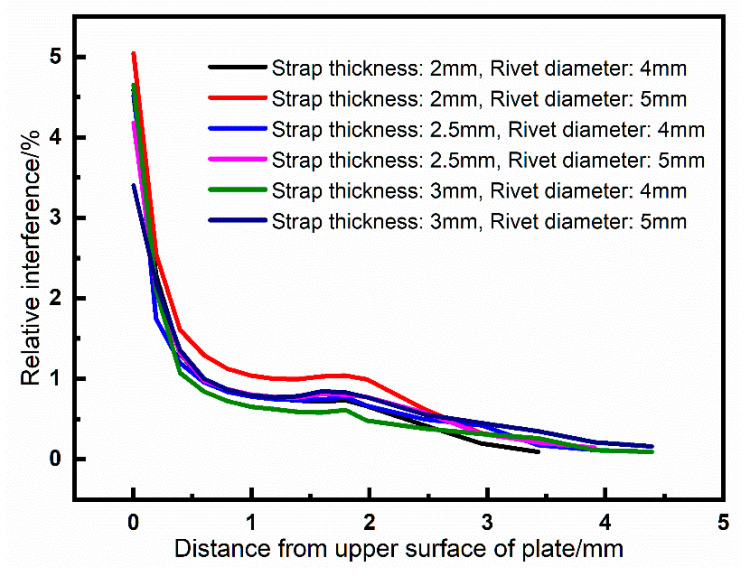
Interferences along the selected path of riveted joints. The path is started from the driven head side to the rivet head side. The *X*-axis is the distance from the upper surface of plate to the lower surface of the strap. The *Y*-axis is the relative interference expressed in Equation (2).

During the riveting process, the rivet shank expands in the radial direction and extrude with plate, then the interference is formed. A path is selected from along the axial direction of the hole wall from the driven head side to the rivet head side. The amount of interference along the path is outputted in Figure 10.

As can be seen from Figure 10, the interference is much larger at the driven head side, this is because when the driven head is forming, the extrusion at the driven head side is the largest, and it decreases sharply with the increase of distance. The interference is stable in the range of 0.4–2.5 mm and becomes smaller on the rivet head side. Furthermore, the smaller the strap thickness and the larger the rivet, the greater the interference. The interference has a significant effect on the residual stresses distribution around the riveted hole. The circumferential compressive stress increases with the increase of interference, and the plastic zone will also expand.

#### 3.2.3. Analysis of Residual Stresses Distribution

**Figure 11:**
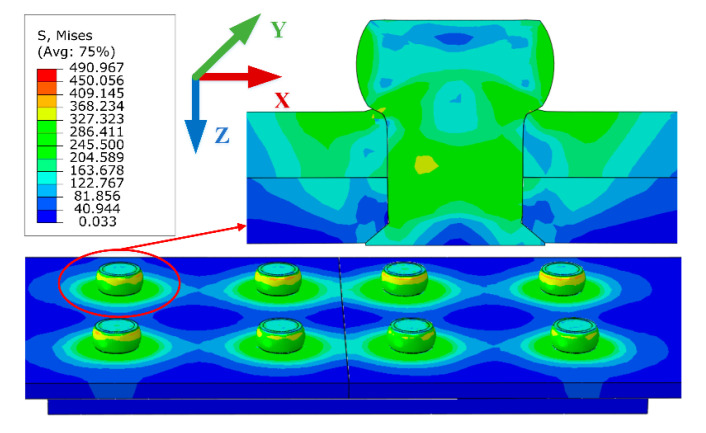
The Von Mises stress of the riveted butt joint after riveting (group 2-5-4).

**Figure 12:**
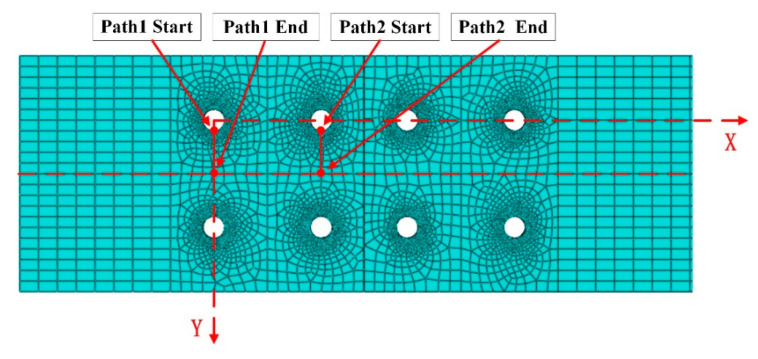
Two paths between adjacent rivets for residual stress distribution investigation.

The residual stress distribution of the riveted joint after riveting is shown in Figure 11. It can be seen that the stress is mainly concentrated in the contact area between rivet and plates. The residual stresses are distributed inhomogeneously along the axial direction of rivet shank, the stress on the driven head side is greater than that on the rivet head side, and the residual stress away from the riveted hole is almost zero. The stress concentration of the riveted joint is easy to occur in the riveted hole area. The fatigue failure may be located at the connection position of two rivets. Therefore, two paths are selected as shown in Figure 12 to investigate the residual stresses distribution around the rivets.

**Figure 13:**
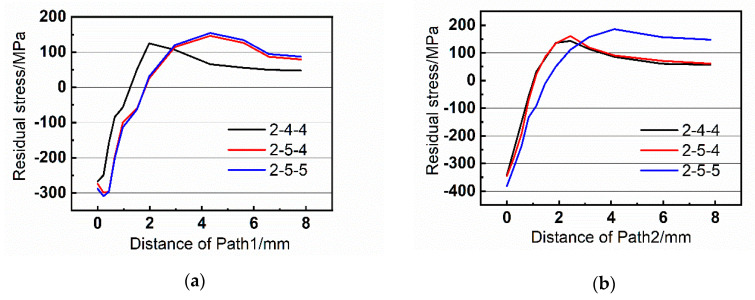
Residual stress of the paths with strap thickness of 2 mm. (**a**) Path1 and (**b**) Path2.

**Figure 14:**
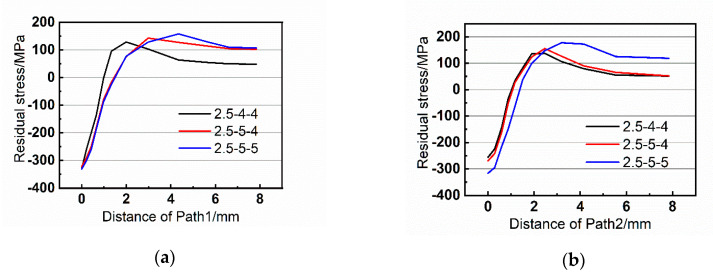
Residual stress of the paths with strap thickness of 2.5 mm. (**a**) Path1 and (**b**) Path2.

**Figure 15:**
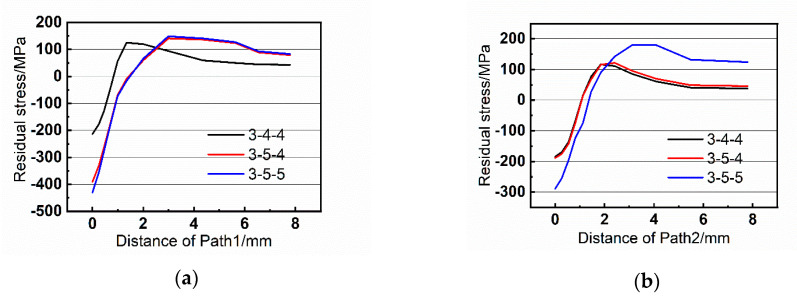
Residual stress of the paths with strap thickness of 3 mm. (**a**) Path1 and (**b**) Path2.

Figure 13, Figure 14 and Figure 15 exhibit the residual stress distributions of the nine groups of riveted joints. It can be demonstrated that the residual stress is compressive near the hole wall, which helps to restrain the fatigue crack initiation of riveted joints. However, when the distance increases, the residual stress becomes tensile stress, and the stress tends to zero when it is far from the hole wall. 

Moreover, when the rivet diameter is 5 mm, the residual stress is larger than that with 4 mm, and the area of compressive stress is also increased. For example, comparing the group 2-4-4 and 2-5-5 in Figure 13a, the residual stress becomes tensile at 1.2 mm in group 2-4-4 while the turning point in 2-5-5 is 1.75 mm. Therefore, the increase of rivet diameter is beneficial to improve the fatigue performance of riveted butt joints.

**Figure 16:**
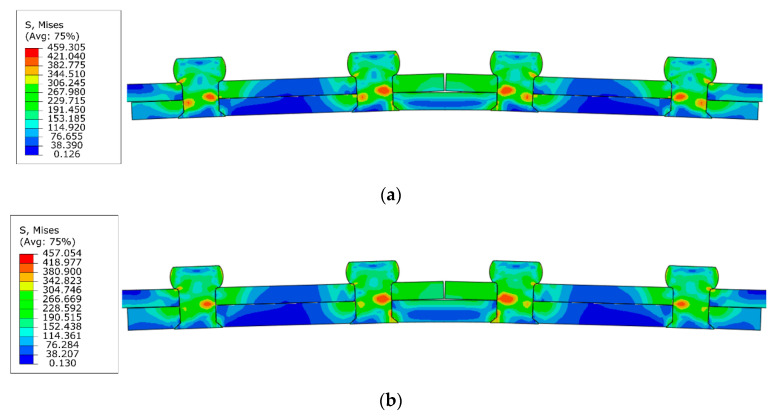

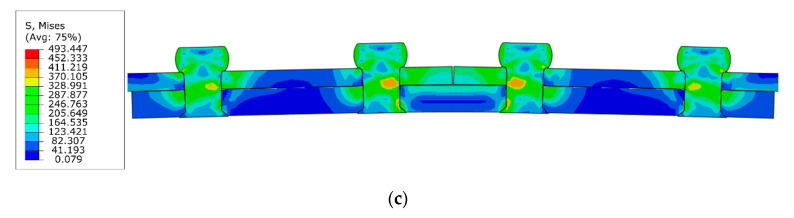
The Von Mises stress of the riveted butt joints under tensile load. The second kind of bending effect can be seen clearly in the diagrams, which will reduce the fatigue performance of the riveted joint. (**a**) Group 2-4-4; (**b**) Group 2.5-4-4; and (**c**) Group 3-4-4.

The residual stress distributions of the riveted butt joints under tensile load are illustrated in Figure 16. The second kind of bending effect can be seen clearly in the diagrams. The bending will cause additional bending distance when the joint is subjected to the tensile load, which will shorten the fatigue life of the riveted joint. When the thickness of the strap increases, the second bending effect of the joint will be weakened so the fatigue performance will be improved.

## 4. Experiments

The fatigue tests are carried out in this section to verify the simulation analysis results and further study the influence of strap thickness and rivets sizes/arrangements on the fatigue performance of riveted joints.

### 4.1. Experimental Procedure

#### 4.1.1. Specimen Production

**Figure 17:**
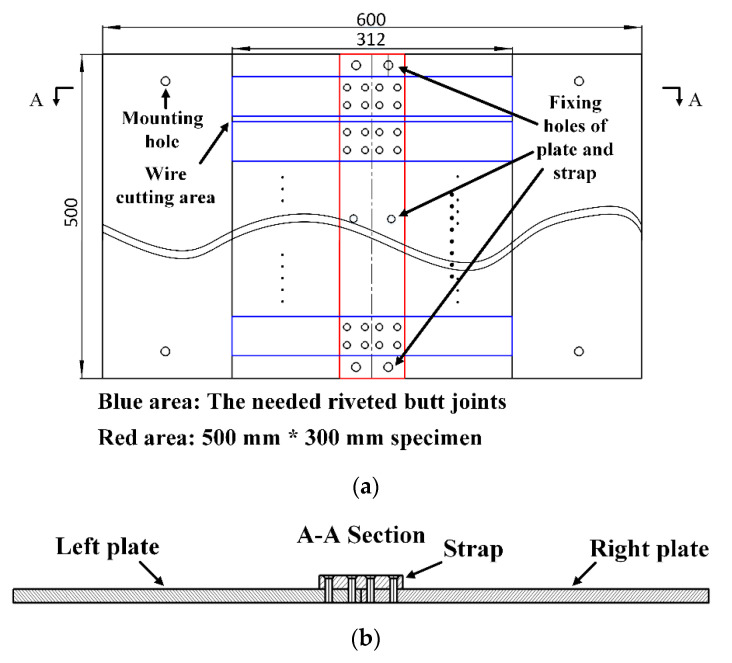
The double-riveted single strap joint specimen. (**a**) Top view and (**b**) side view.

**Figure 18:**
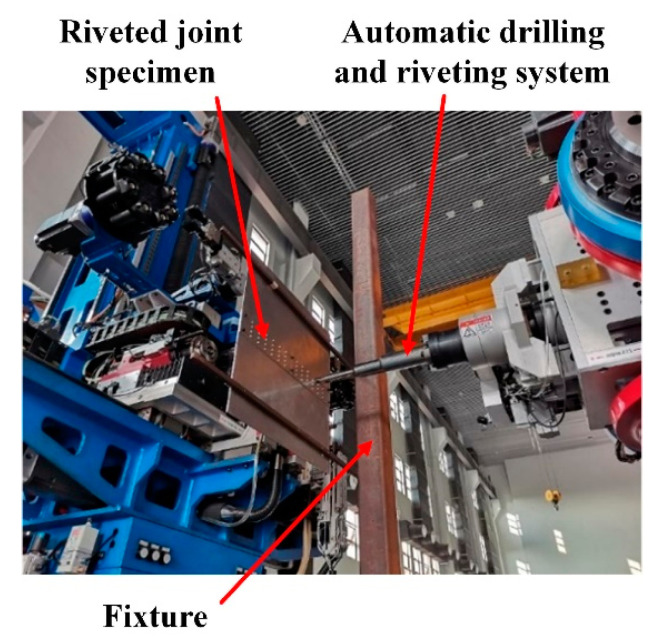
The riveted joints specimen production by automatic drilling and riveting system.

The size of the initial riveted specimen is 500 mm×312 mm, as shown in Figure 17. The material of straps and plates is 2024-T3 aluminum alloy and the material of YSA622-100° rivets is 2117-T4 aluminum alloy. The riveted joints specimen was fixed on the fixture and processed by the automatic drilling and riveting system, as shown in Figure 18. After riveting is completed, the redundant parts of the plates were removed by wire cutting.

The driven head geometries after riveting were measured and compared with simulation results. The measured data are averaged and shown in Table 6. The comparative results indicate that the error between simulation and experimental results is within 5%, so the FE model can be considered to be corrective and effective.

#### 4.1.2. Static Tensile Tests

**Figure 19:**
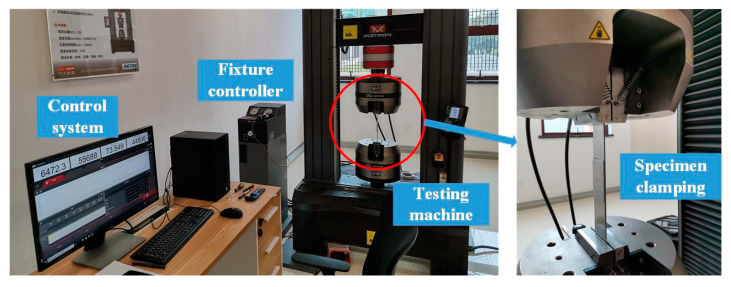
The static tensile tests using INSTRON 5985 universal testing machine.

As shown in Figure 19, the static tensile tests were carried out using an INSTRON 5985 universal testing machine (Instron Corporation, Canton, America). Three specimens of group 2-4-4 are used to obtain the basic static failure load $F_{b}$. The specimen is clamped by the fixtures at the clamping areas on the left and right plates. The tensile load is applied until the specimen breaks, the load recorded at the breaking time is the static failure load $F_{b}$. The test results are shown in Table 7.

#### 4.1.3. Fatigue Tests

**Figure 20:**
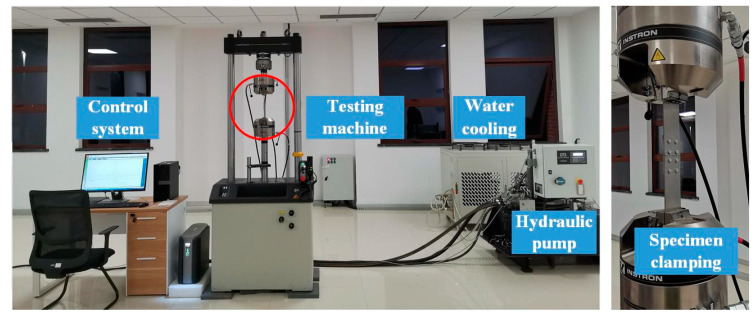
The fatigue tests using INSTRON 8801 fatigue testing machine.

The specimens were fatigue tested with an INSTRON 8801 fatigue testing machine (Instron Corporation, Canton, America) as shown in Figure 20. The constant amplitude load of tension situation is applied to all riveted specimens. The maximum load is set as $F_{max}=0.7\times F_{b}=8.451\;\mathrm{KN}$ and the load amplitude is 3.803 KN. The fatigue tests have been performed under a frequency of 10 Hz and a stress ratio of 0.1. Three specimens are tested for each group of the riveted joints. The test temperature is regarded as constant. The experimental results of crack sites and fatigue lives will be presented and discussed in the next section.

### 4.2. Experimental Results and Discussion

The fatigue lives and cracks site results are illustrated in Table 8.

**Figure 21:**
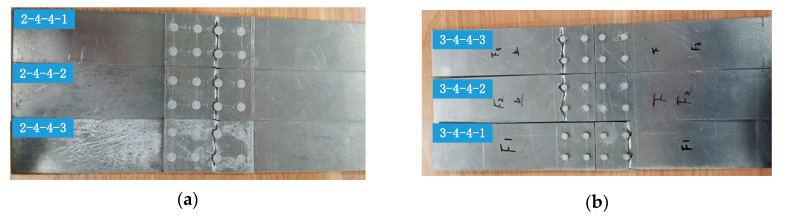
Typical fatigue crack morphology of specimens. (**a**) Three specimens of group 2-4-4, crack sites are on straps and (**b**) three specimens of group 3-4-4, crack sites are on plates.

Some of the typical fatigue crack morphologies on straps and plates are shown in Figure 21, combining with the statistics of the crack sites in Table 8, the following results can be obtained:

1. The fatigue cracks of the riveted butt joints are taken place on straps or plates, there is no shear failure of rivets. Therefore, the failure mode of the joints is mainly the tensile failure of the strap or plate. When the strap thickness is the same as the plate thickness, the crack site is on the strap. When the strap thickness is larger than the plate thickness, the crack site is on the plate.
2. When the strap thickness is 2 mm, the crack sites occurred at the 2nd or 3rd column of rivets on the straps, and the 3rd column has a higher probability (77.8%). When the strap thickness is greater than 2 mm, the crack sites occurred at the 1st or 4th column of rivets, and the probability of the 4th column is larger (61.1%).

**Figure 22:**
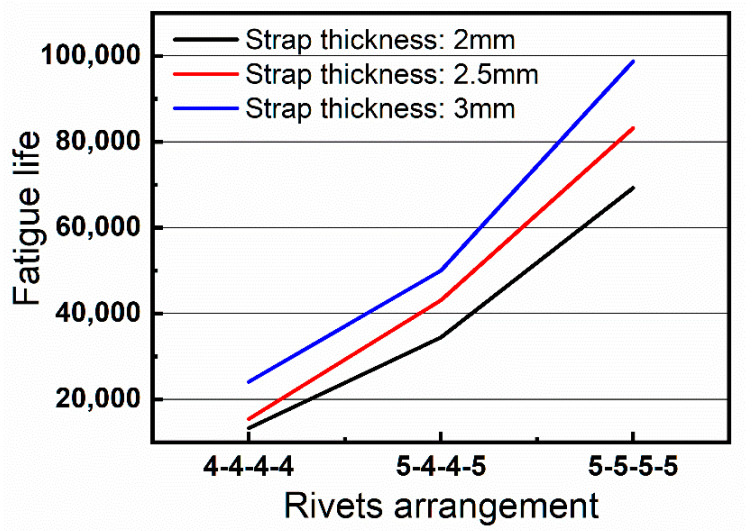
The fatigue lives of different groups of riveted butt joints with various strap thicknesses and rivets sizes.

The relationships between fatigue lives of riveted joints and the strap thickness and rivets sizes are shown in Figure 22. It should be noticed that the *X*-axis represents the rivets arrangements, as an example, the 5-4-4-5 represents that the diameter of the 1st and 4th columns of rivets is 5 mm, the 2nd and 3rd is 4 mm. The *Y*-axis is the average fatigue life of the riveted joints.

The following results can be demonstrated in Figure 22:

1. Take group 2-4-4 as an example, if the diameter of 1st and 4th columns of rivets is changed from 4 mm to 5 mm, the fatigue life of the riveted joint will increase by 16.4%. Moreover, if all the four columns are replaced with 5 mm rivets, the fatigue life of the riveted joint is increased by 81.4%. The same increase can also be seen in the riveted joints with strap thicknesses of 2.5 mm and 3 mm. Therefore, the increase of strap thickness can significantly increase the fatigue life of riveted joint, this is consistent with the simulation analysis results.
2. When the rivets arrangement is 5-4-4-5, comparing with the 2 mm thick strap, the fatigue lives of the riveted joints are increased by 179% and 438%, respectively, when the strap thickness is 2.5 and 3 mm. This result indicates that increasing rivet size is highly beneficial to the fatigue performance of the riveted butt joint, this also proves the validity of the simulation results.

The experimental results verified the analysis results of the residual stresses distribution in FE model. In a word, increasing the rivet size and strap thickness is a practical and effective way to enhance the fatigue performance of the riveted butt joint. Furthermore, the cyclic temperature load has a great impact on the residual stresses and fatigue life of riveted joints, and that will be the focus of our research in the next stage.

## 5. Conclusions

Riveting is one of the most important connection methods in aircraft assembly. The connection quality and fatigue performance of riveted joints directly affect the flight reliability and service time of aircraft. In this paper, under the assumption of constant temperature, the residual stresses distribution of riveted single strap butt joints are numerically studied with various rivet sizes and strap thicknesses. The fatigue tests with cyclic loading are carried out to investigate the fatigue life of riveted butt joint specimens with various parameters. The suggestions for structural design parameters optimization of the riveted butt joint are proposed. The conclusions of this paper are listed as follows:

1. The residual stress near the riveted hole is compressive, which can reduce the local stress concentration and improve the fatigue performance of riveted joints.
2. Increasing the size of the rivets can significantly improve the fatigue life of the riveted butt joint. For group 2-4-4, increasing the diameter of the first and fourth columns of rivets from 4 to 5 mm can increase the fatigue life by 16.4%. Moreover, when all rivets are changed to 5 mm, the fatigue life of the riveted butt joint is increased by 81.4%.
3. Increasing strap thickness is beneficial for the fatigue performance enhancement of riveted butt joints. When the rivets arrangement is 5-4-4-5 was compared with a 2 mm thick strap, the fatigue life is increased by 179% for 2.5 mm strap and 438% for 3 mm strap.

## Figures and Tables

**Table 1:** The geometries of riveted single strap butt joint in different groups (unit: mm).

Group ^1^	Strap Thickness	Rivet Diameter of 1st Column	Rivet Diameter of 2nd Column	Rivet Diameter of 3rd Column	Rivet Diameter of 4th Column
2-4-4	2	4	4	4	4
2-5-4		5	4	4	5
2-5-5		5	5	5	5
2.5-4-4	2.5	4	4	4	4
2.5-5-4		5	4	4	5
2.5-5-5		5	5	5	5
3-4-4	3	4	4	4	4
3-5-4		5	4	4	5
3-5-5		5	5	5	5

^1^ The group names are set as “strap thickness-rivet diameter of 1st column-rivet diameter of 2nd column”, the 3rd and 4th columns of rivets are symmetrical to the first two columns.

**Table 2:** Material properties for 2024-T3 aluminum alloy plates [9].

Parameter of Panel	Value
Young’s modulus	72.4 GPa
Poisson’s ratio	0.33
Initial yield stress	310 MPa
Hardening parameters when ε ≤ 0.02	C = 676 MPa and m = 0.14
Hardening parameters when 0.02 < ε ≤ 0.1	C = 745 MPa and m = 0.164
Slope of linear hardening curve when ε > 0.1	1034 MPa

**Table 3:** Material properties for 2117-T4 aluminum alloy YSA621-100° rivet [9].

Parameter of Rivet	Value
Young’s modulus	71.7 GPa
Poisson’s ratio	0.33
Initial yield stress	172 MPa
Hardening parameters when 0.02 ≤ ε ≤ 0.1	C = 544 MPa and m = 0.23
Hardening parameters when 0.1 < ε ≤ 1.0	C = 551 MPa and m = 0.15

**Table 4:** Displacement loads for different strap thicknesses and rivets (unit: mm).

Strap Thickness	Diameter	Displacement Load
2	4	3.393
	5	3.378
2.5	4	3.124
	5	3.663
3	4	3.410
	5	3.517

**Table 5:** The driven head geometries obtained from simulation results (unit: mm).

Strap Thickness	Rivet Diameter	Diameter of Driven Head		Height of Driven Head
		Standard ^1^	Simulation	
2	4	6.0 ± 0.4	6.19	2.66
	5	7.5 ± 0.5	7.59	2.65
2.5	4	6.0 ± 0.4	6.18	2.43
	5	7.5 ± 0.5	7.68	2.88
3	4	6.0 ± 0.4	6.22	2.64
	5	7.5 ± 0.5	7.72	2.52

^1^ The standard values of the driven head diameter refer to [25].

**Table 6:** The driven head geometries obtained from simulation results (unit: mm).

Rivet Diameter	Diameter of Driven Head			Height of Driven Head		
	Simulation	Experiment	Error	Simulation	Experiment	Error
4	6.20	6.09	1.78%	2.58	2.65	3.10%
5	7.66	7.47	2.48%	2.68	2.81	4.85%

**Table 7:** The tests result of static tensile failure load $F_{b}$ (unit: KN).

Group	Specimen 1	Specimen 2	Specimen 3	Average
2-4-4	12.018	12.128	12.067	12.071

**Table 8:** The fatigue lives and cracks sites of various riveted butt joints from fatigue tests.

Group	Specimen	Fatigue Life	Average	Crack Site
2-4-4	1	12,621	13,280	3rd column of rivets on strap
	2	13,858		3rd column of rivets on strap
	3	13,362		3rd column of rivets on strap
2-5-4	1	16,508	15,463	3rd column of rivets on strap
	2	14,994		2nd column of rivets on strap
	3	14,887		2nd column of rivets on strap
2-5-5	1	22,554	24,094	3rd column of rivets on strap
	2	24,389		3rd column of rivets on strap
	3	25,340		3rd column of rivets on strap
2.5-4-4	1	34,362	34,408	4th column of rivets on plate
	2	33,881		1st column of rivets on plate
	3	34,981		1st column of rivets on plate
2.5-5-4	1	42,886	43,098	1st column of rivets on plate
	2	44,887		4th column of rivets on plate
	3	41,522		1st column of rivets on plate
2.5-5-5	1	49,891	50,001	4th column of rivets on plate
	2	47,957		4th column of rivets on plate
	3	52,154		1st column of rivets on plate
3-4-4	1	68,853	69,271	4th column of rivets on plate
	2	69,488		1st column of rivets on plate
	3	69,473		1st column of rivets on plate
3-5-4	1	84,453	83,183	4th column of rivets on plate
	2	83,243		4th column of rivets on plate
	3	81,853		4th column of rivets on plate
3-5-5	1	99,260	99,181	4th column of rivets on plate
	2	98,812		4th column of rivets on plate
	3	99,473		4th column of rivets on plate
